# Creating an amateur press corps of graduate students and postdoctoral fellows to cover breaking science and improve lay-writing skills

**DOI:** 10.1017/cts.2021.829

**Published:** 2021-07-30

**Authors:** Kimberly McGhee, Tammy Loucks, Sheila Champlin, Matthew Greseth, Paula Traktman

**Affiliations:** 1 South Carolina Clinical and Translational Research Institute, College of Medicine, Medical University of South Carolina, Charleston, SC, USA; 2 College of Graduate Studies, Medical University of South Carolina, Charleston, SC, USA; 3 Academic Affairs Faculty, Medical University of South Carolina, Charleston, SC, USA; 4 Office of Communications and Marketing, Medical University of South Carolina, Charleston, SC, USA

**Keywords:** Science communications, science writing, internship, lay public, general public, graduate education, interdisciplinary, career development, plain language

## Abstract

The Science Writing Initiative for Trainees is a science communications internship program for biomedical graduate students and postdoctoral fellows at the Medical University of South Carolina. Interns serve as an amateur press corps, writing news stories and press releases about recent high-impact research articles. Since 2016, 25 interns have written more than 100 EurekAlert! press releases that have received more than a half million views. Interns learn to explain science across the translational spectrum and to convey findings in plain language to a lay audience, serving as ambassadors for science.

## Introduction

The importance of communicating science to a general audience has been driven home by the COVID-19 pandemic, when it is absolutely critical that the public understands and trusts science, and when the perils of its failing to do so have become all too clear [1–4]. Long before the pandemic, however, the need for improved science communication to the lay public and policy makers had been recognized [5–7] and incorporated into key competencies for biomedical [8], translational, and clinical researchers [9, 10]. The success of interdisciplinary collaborations, increasingly common in science and essential to translational research, also hinges in part on the ability of group members to communicate outside the jargon of their own specialty [11, 12]. Clinical researchers also need to communicate to patients, both during the recruitment for clinical trials but also in communicating the results of those trials back to patients [13]. Without the ability to tailor their language and frames of reference to those of their intended audience, scientists will struggle to achieve these aims.

Integrating science communication training into an already crowded biomedical science curriculum can be challenging. One-time workshops are rarely effective, as students are not given sufficient time to practice the newly taught skills. Students need time to develop the habit of writing [14], and rewriting, ideally with feedback from an experienced mentor. Experiential learning in science communications could offer a solution, providing graduate students “on-the-job” training and the opportunity to complete real-world writing assignments [15]. However, finding time away from the laboratory for a months-long internship can present a barrier to graduate students or postdoctoral fellows [16], who do not wish to cut their research productivity but are interested in developing science communication skills.

The Medical University of South Carolina (MUSC) has launched a successful and flexible internship, SC-SWIFT (South Carolina Science Writing Initiative For Trainees), which offers its biomedical graduate students and postdoctoral fellows much-needed training in science communications through a collaboration between the College of Graduate Studies (CGS), the Office of Communications and Marketing (OCM) and the South Carolina Clinical and Translational Research Institute (SCTR), a Clinical and Translational Science Awards (CTSA) hub with an academic home at MUSC. Interns accept assignments on a project-by-project basis, as their schedules permit, and work with a professional science writer, now embedded in CGS and SCTR, to create news stories and press releases about the latest research from the university.

## Materials and Methods

In 2016, the CGS and the OCM began to offer postdoctoral fellows the opportunity to write news releases about recent MUSC research for EurekAlert! (https://www.eurekalert.org/), the science news site run by the American Association for the Advancement of Science. Interns were not allowed to write on research from their own laboratories to avoid any appearance of conflict of interest. Interested fellows were invited to a four-session science communications boot camp, where they learned what makes an article “newsworthy” and practiced a variety of techniques for writing for a lay public. The boot camp, which was led by the professional science writer who directs the internship, consisted of four 2-hour sessions over the course of two weeks and was attended by more than a dozen postdoctoral fellows. Six boot camp participants opted to go forward with the internship, after receiving the permission of their mentors. All six interns published releases on recent high-impact MUSC research, and three continued to write EurekAlert! releases regularly and to contribute stories to MUSC’s medical magazine.

In 2018, the internship was expanded to graduate students, who were invited to participate in the boot camps. Interest in the internship was especially strong among graduate students selected for the T32 training grant, titled “Cellular, Biochemical and Molecular Sciences Training Program: Developing the skills and expertise needed for a changing biomedical landscape” (principal investigator: Dr. Paula Traktman), which was awarded to the CGS in 2019. The program offers trainees the opportunity to focus in two of four areas of concentration: science communications, entrepreneurship, science education, and community engagement/advocacy. Seven (87.5%) of the eight T32 students have thus far opted for the concentration in science communications.

In early 2020, the CGS Speaks blog was launched and was intended to give CGS students and postdoctoral fellows a platform for sharing stories about science as well as their experiences as young scientists. The blog also provides interns with an additional writing outlet, where they can discuss scientific advances more broadly without having to restrict themselves only to MUSC research, as with EurekAlert! releases. It is managed by a second writer, brought on in late 2020, who also serves as a mentor to interns as they write news stories and releases.

The two embedded writers help interns to identify recent, newsworthy MUSC research. They prioritize for coverage recent articles published in high-impact journals that feature MUSC faculty as lead or senior authors and/or articles that provide information likely to be of relevance to a broader audience. They learn about publications from publishers, researchers, MUSC’s public relations office, and PubMed searches. Interns decide which of these stories they will cover and always have the option to consult PubMed themselves and suggest a release of their own or to pitch an idea for a news story to the MUSC digital news editor, as some did during the pandemic. The intern directors work closely with public relations to avoid duplication of stories.

The embedded writers also work with the interns, particularly newer ones, throughout the drafting of EurekAlert! releases. They accompany them to interviews with the authors of research articles, review two to three drafts of the release to ensure that the content is accurate and understandable for a lay audience, and arrange for photographs or other artwork to accompany the releases. As interns become more experienced, less supervision is needed, but the draft of the release is always reviewed by a professional writer. Once the writer and the intern have finalized the draft, the intern sends it to the stakeholder for review and incorporates any requested changes before the writer posts it to EurekAlert!.

EurekAlert! releases are picked up by national news agencies and feeds, and we track the underlying scientific manuscript’s Altmetric score [17], a measure of the media and social media attention it is receiving, using the free Altmetric bookmarklet (https://www.altmetric.com/products/free-tools/bookmarklet/). The releases are repurposed through a variety of communications avenues, including MUSC’s news center and medical magazine, as well as websites for SCTR, the Office of Innovation, and the Hollings Cancer Center, providing the interns professional bylines. Interns are encouraged to create an e-portfolio of their published works, to which they can link from their professional online profiles.

To document interns’ perceptions of the program and learn about how they would like to see it develop, we sent a REDCap [18] survey in 2020 to 16 previous or current interns, asking them to respond anonymously to a series of statements about the internship program using a five-point Likert scale ranging from “strongly agree” to “strongly disagree.” They were also given an opportunity to provide comments.

## Results

**Fig. 1.**
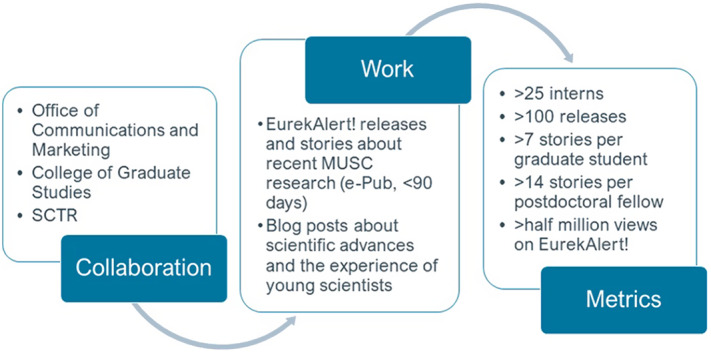
Workflow and metrics for the South Carolina Science Writing Initiative For Trainees (SC-SWIFT), a science communications internship program at the Medical University of South Carolina (MUSC). SCTR = South Carolina Clinical and Translational Research Institute.

Since its inception, more than 18 graduate students (seven of whom were T32 trainees) and nine postdoctoral fellows have participated in the internship, writing about science across the translational spectrum and in their own as well as other disciplines. On average, graduate students completed 7.3 stories and remained in the internship for 2 years, while postdoctoral fellows completed 14.8 stories and remained for 3.2 years. The most productive interns were those considering a transition to a science communications career. This held true for both the top-producing graduate student (22 stories) and postdoctoral fellow (48 stories). Collectively, interns have posted over 100 releases, which have received more than a half million views on EurekAlert! (Fig. 1).

**Fig. 2.**
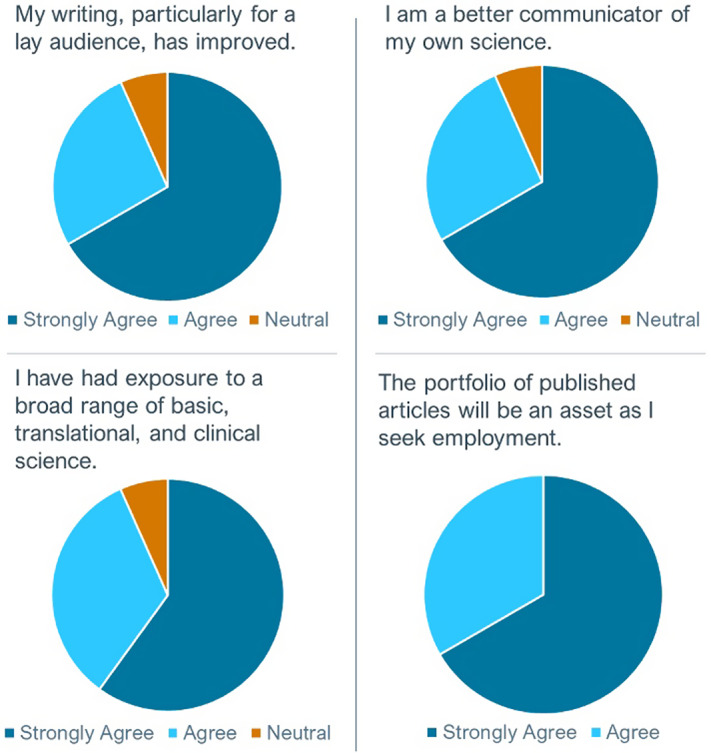
Survey results for 15 present or past South Carolina Science Writing Initiative For Trainees (SC-SWIFT) interns.

Survey results show that interns see the value of the program (Fig. 2). Of the 16 interns surveyed about the program, 15 (93.8%) responded. Eight (53.3%) were students and six (40%) were fellows. Of the respondents, more than 93% strongly agreed (67%) or agreed (27%) their science communication skills had improved and strongly agreed (60%) or agreed (33%) that they had been exposed to a broad range of basic, translational, and clinical science. All strongly agreed (67%) or agreed (33%) their writing portfolio would be helpful for their career.

Indeed, students believe the training, real-world experience, and published bylines aid them in their career search, whether inside or outside academia. Five interns are now working full-time in some facet of science writing or communications, and all of them found having an e-portfolio of published works helpful in their career transition.

The survey also invited comments from interns on the strengths of the program and on areas that need improvement. Interns commented that they “liked being able to excite others about scientific work” and “enjoyed talking with scientists across a broad range of specialties.” Interns also commented on their improved communication skills, with one noting that “communicating science to a lay audience is easier now, and my technical writing is clearer.” To improve the program, interns suggested more opportunities for interns to network with one another and with science writers in the region and more career development resources. Additional career development resources would also support trainees’ desire to explore alternative careers.

The program has benefited not only the interns but also the university by helping raise the profile of its researchers. Releases posted to EurekAlert! are frequently picked up by news outlets and shared via digital feeds. As a result, the Altmetric score [17] of an article often increases dramatically after a release, often from 0 or single digits to an average of 30–50. The Altmetric score for many articles goes much higher – with the highest for an intern-produced release in 2020 being 331, with pickups by 36 news outlets and eight blogs.

The internship has also helped to inform the community about science crucial to public health. During the pandemic, interns successfully “pitched” their own COVID-19 news stories to the OCM, including Q & As with local experts willing to share their knowledge. One intern interviewed a noted MUSC virologist, including questions about masking and social distancing. That Q & A received over 29,000 views, was shared widely on social media, and was the second most-read article of 2020 for MUSC’s news center.

## Discussion

Traditional graduate training in the biomedical sciences has sometimes risked constraining students into a narrow specialty. Students eager to prove their competence in their newfound specialty adopted its highly specific language but in doing so began to lose the ability to communicate that science more widely. The SC-SWIFT internship reminds them that they must articulate that science for diverse audiences if they are to be successful scientists. It provides them the opportunity both to discuss science with those outside their specialty, a skill that will be crucial to forming successful cross-disciplinary collaborations for the future, and to translate those findings for a general public. The internship has also provided participants a more robust knowledge of the translational spectrum. Interns whose own work is focused more on the basic sciences write news stories and releases on findings from translational and clinical researchers, helping them to understand what is required for data to be clinically meaningful.

Finding the time in an already crowded graduate curriculum or away from a busy research lab has sometimes been a barrier to science communication initiatives [19]. By remaining flexible and assigning interns projects on a one-by-one basis, as their schedules permit, the program has enabled interns to hone their lay-writing skills without impinging on their laboratory responsibilities.

An unexpected benefit of the program has been the opportunity it gives to postdoctoral fellows and graduate students who are lead authors of an article being profiled to discuss their findings in a lay-friendly way. The intern typically interviews the senior author of a publication and, when possible, the lead author, who in many cases is a graduate student or postdoctoral fellow, and includes quotations by both in the final news story or release. The exercise not only offers the intern the opportunity to learn about high-impact research outside his or her own field but it also offers the graduate student or postdoctoral fellow being interviewed the opportunity to comment on his or her findings and their significance in a lay-friendly way, all in the presence of the senior author who can suggest tweaks in the explanation where necessary. A number of these senior authors/mentors have commented that this opportunity was a very valuable learning experience for their students or postdoctoral fellows.

For those interested in pursuing a career in science writing, the internship offers an opportunity to build an e-portfolio of published works that showcases their skills. Of the 25 or so graduate students and postdoctoral fellows who have participated in the internship thus far, five have transitioned to full-time careers in science writing or communications in industry. Many interns felt that having a portfolio of published work provided them the edge they needed in navigating a career transition to this new field.

## Next Steps

Within the next year, the program would like to start offering digital badges [20] in science communications, toward which the news stories, releases, and blog posts will count. Interns will be able to post the digital badge to their social media channels to document their science communications training. Once pandemic restrictions have eased, we plan to bring science writers in a variety of fields (journalists, publications writers, regulatory writers), including alumni of the internship program, to networking events on campus. In addition to surveying the interns periodically, we hope to survey others impacted by the program, including mentors, the professional writers working with the interns, the researchers whose work has been profiled in the releases and news stories, and university communicators. We also hope to have lay focus groups read selected releases to assess how well they have understood the science and to pinpoint ways to improve accessibility.

## Conclusion

SC-SWIFT, a collaboration between the CGS, CTSA program hub, and the OCM at MUSC, is a flexible internship program that has offered over 25 graduate students and postdoctoral fellows the opportunity to hone their lay-writing skills by serving as an amateur science press corps, creating and publishing more than 100 EurekAlert! releases that have received more than a half million views internationally. The program could be reproduced at other universities by forming interdisciplinary collaborations between CTSA hubs or Colleges of Graduate Studies and English/Communications Departments or public relations offices. Interns learn to explain science across disciplinary boundaries and the translational spectrum as well as to convey findings in plain language to a lay audience, improve their writing skills through mentoring across multiple assignments, and serve as ambassadors for science to the wider community.
